# Seasonal Variation of *Cistus ladanifer* L. Diterpenes

**DOI:** 10.3390/plants1010006

**Published:** 2012-07-26

**Authors:** Juan Carlos Alías, Teresa Sosa, Cristina Valares, José Carlos Escudero, Natividad Chaves

**Affiliations:** Department of Plant Biology, Ecology and Earth Sciences, Faculty of Science, University of Extremadura, 06080 Badajoz, Spain; Email: tesosa@unex.es (T.S.); cvalmas@unex.es (C.V.); escudero@unex.es (J.C.E.); natchalo@unex.es (N.C.)

**Keywords:** *Cistus ladanifer* L., secondary metabolism, diterpenes, temperature, water stress

## Abstract

The exudate of *Cistus ladanifer* L. consists mainly of two families of secondary metabolites: flavonoids and diterpenes. The amount of flavonoids present in the leaves has a marked seasonal variation, being maximum in summer and minimum in winter. In the present study, we demonstrate that the amount of diterpenes varies seasonally, but with a different pattern: maximum concentration in winter and minimum in spring-summer. The experiments under controlled conditions have shown that temperature influences diterpene production, and in particular, low temperatures. Given this pattern, the functions that these compounds perform in *C. ladanifer* are probably different.

## 1. Introduction

**Figure 1:**
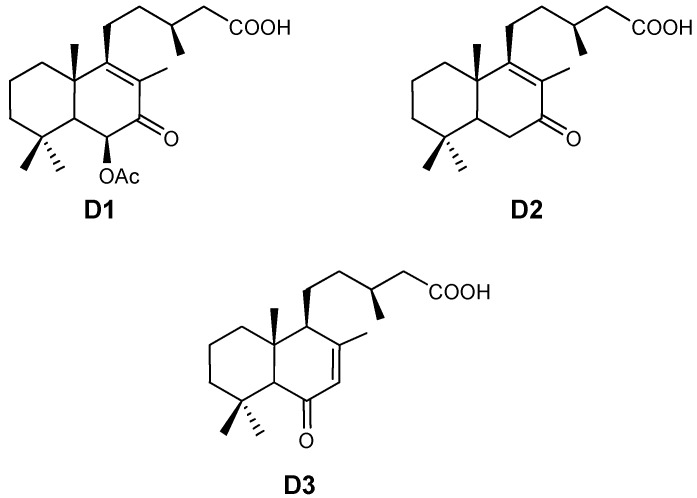
Chemical structures of diterpenes in the exudate of *C. ladanifer*.

*Cistus ladanifer* L. (rock-rose or jara) is a typical Mediterranean species [1], widely distributed over Western Spain. Leaves and stems secrete an abundant exudate of secondary metabolites [2,3]. The exudate of *C. ladanifer* is composed fundamentally of compounds of phenolic and terpene origin [4]. Phenolics and terpenoids are involved in many plant processes, particularly those responding to environmental biotic and abiotic stimuli. Bell [5] proposed that the synthesis of these compounds should be regarded as a defense mechanism of the plant against stress [6,7,8,9]. Previous studies have shown that the phenolic compounds synthesized by *C. ladanifer*, in particular six aglycone flavonoids, make up between 0.6% and 3.4% (depending on the season) of the dry weight of the leaf [10]. Their synthesis is markedly seasonal: they are the majority products in summer, but in winter their presence is minimal [2,11]. On the other hand, Alías [12] has demonstrated that in the exudate of *C. ladanifer* the majority terpenes are three diterpenes (D1: 6-acetoxy-7-oxo-8-labden-15-oic acid; D2: 7-oxo-8-labden-15-oic acid; D3: oxocativic acid). The compounds obtained were identified by comparing their spectral characteristics by NMR with those described in the literature [13,14,15]. Figure 1 shows the molecular structure of the compounds. In addition, it has been demonstrated their presence could be involved in the allelopathic activity of this specie [12,16,17].

The great ecological interest in plant terpenoids is reflected in several reviews of their ecological chemistry and role [18,19]. Although changes in plant terpene concentrations and emission rates are of great ecological interest, little is known of the factors controlling terpene concentrations.

In particular, the objective was to assess how terpenoid levels vary naturally over a long period of time, and to evaluate the environmental factors responsible for their synthesis to determine and inquire more deeply into the possible role that these compounds are playing.

## 2. Results and Discussion

### 2.1. Seasonal Variation

Table 1 presents the amounts in mg/g dry-wt of each of the diterpenes analyzed in the leaves samples of *C. ladanifer*. The results for the different seasons correspond to the mean values of the 4 populations studied over two years. It can be observed that in the leaves there were statistically significant variations in diterpene amount between the seasons (ANOVA; *p* < 0.05). It was also in winter when the differences between them were greatest, with practically double the amounts of D1 and D3 being synthesized (8.03 and 8.78 mg/g dry-wt, respectively) relative to D2 (4.10 mg/g dry-wt). The synthesized amounts of D1 and D2 were similar during spring, summer, and autumn, whereas the lowest values for D3 corresponded to summer and autumn (3.25 and 3.56 mg/g dry-wt, respectively), doubling in spring (6.51 mg/g dry-wt), and reaching the maximum in winter (8.79 mg/g dry-wt). The present results show that the amount of the diterpenes constituting the exudate of *C. ladanifer* is clearly greater in winter, with clear and significant differences relative to the other seasons.

**Table 1:** Amounts (mg/g dry-w) of diterpenes (D1: diterpene 1; D2: diterpene 2; D3: diterpene 3) in leaves collected in autumn, winter, spring, and summer in the selected populations. The values are the means of the four populations studied over two years. SD: standard deviation.

		Spring	Summer	Autumn	Winter	ANOVA
**Leaves**	**D1**	3.50 ^a^	3.28 ^a^	3.33 ^a^	8.03 ^b^	*p* < 0.05
	SD	0.73	2.04	1.04	0.77	
	**D2**	2.76 ^a^	2.03 ^a^	2.38 ^a^	4.10 ^b^	*p* < 0.05
	SD	1.09	1.03	0.67	0.64	
	**D3**	6.51 ^b^	3.25 ^a^	3.56 ^a^	8.79 ^c^	*p* < 0.05
	SD	2.36	0.98	1.51	3.11	
	**Total diterpenes**	12.78 ^a^	8.57 ^a^	9.28 ^a^	20.93 ^b^	*p* < 0.05

^a, b, c^: Same letter denote the absence of statistical differences (Tukey test).

There are very few studies that quantify the amount of diterpenes in Mediterranean shrubs. Most studies are focused on quantifying the emissions of aromatic terpenes such as monoterpenes [20] or on the quantification of total terpenes [21]. In any case, other Mediterranean species such as *Rosmarinus officinalis* shows a behavior similar to that of *C. ladanifer*. Thus, the highest concentrations of the major diterpenes carnosic acid and carnosol were found during the winter and the lowest concentrations during the summer [22]. These results are in accordance with the observations of Levinshon *et al*. [23]. Seasonal variation in the presence of diterpenes suggests that the synthesis of these compounds could be induced by seasonal climatic factors such as temperature and water stress.

### 2.2. Temperature and Water Stress

Table 2 shows the statistical analysis of the results (two-way ANOVA) of the trials carried out under controlled conditions. This test quantified the percentage variations in the amount of diterpenes from the beginning to the end of each trial. The variations found in the amount of D1 and D2 are explained both by the temperature as water stress. The variations found in the D3 are only explained by temperature.

**Table 2:** Statistical analysis of the results (two-way ANOVA) to test the influence of temperature and water stress (independent variables) on the amount of diterpene in the leaves (dependent variable).

**D1**	**Type III Sum of Squares**	**df**	**Mean Square**	**F**	**Sig.**
Corrected Model	51659.677 ^a^	3	17219.892	3.756	0.025
Intercept	632425.504	1	632425.504	137.955	0.000
Temperature	31066.47	1	31066.47	6.777	0.016
Stress	20447.496	1	20447.496	4.46	0.046
Temperature * Stress	440.874	1	440.874	0.096	0.759
^a^: R Squared = 329 (Adjusted R Squared = 241); *: Interaction between the two variables.					
**D2**	**Type III Sum of Squares**	**df**	**Mean Square**	**F**	**Sig.**
Corrected Model	47756.165 ^a^	3	15918.722	7.45	0.001
Intercept	523104.439	1	523104.439	244.799	0.000
Temperature	33921.278	1	33921.278	15.874	0.001
Stress	13645.168	1	13645.168	6.386	0.019
Temperature * Stress	155.981	1	155.981	0.073	0.789
^a^: R Squared = 493 (Adjusted R Squared = 427); *: Interaction between the two variables.					
**D3**	**Type III Sum of Squares**	**df**	**Mean Square**	**F**	**Sig.**
Corrected Model	47555.955 ^a^	3	15851.985	9.31	0.000
Intercept	383756.337	1	383756.337	225.394	0.000
Temperature	45822.766	1	45822.766	26.913	0.000
Stress	8.728	1	8.728	0.005	0.944
Temperature * Stress	2.305	1	2.305	0.001	0.971
^a^: R Squared = 548 (Adjusted R Squared = 490); *: Interaction between the two variables.					

**Figure 2:**
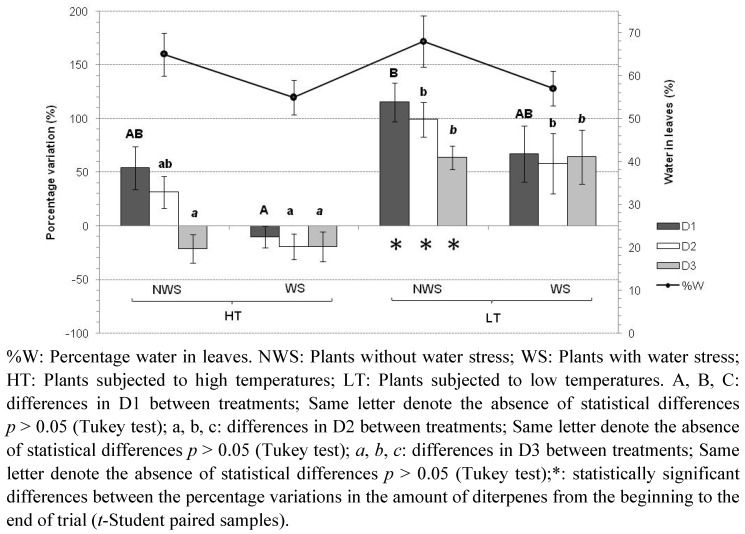
Percentage variation relative to the beginning of the trial in the amount of diterpenes in *C. ladanifer* plants.

Figure 2 shows that there is a clear increase in the amount of the three diterpenes especially under low temperature conditions, and with statistically significant differences for the plants not subjected to water stress.

In particular, under these conditions the amount of diterpene D1 increased by 115%, D2 by 99%, and D3 by 64%. The plants subjected to high temperatures and water stress presented reductions in formation of the three diterpenes but it was not significant. Low temperatures and water stress, however, again led to increased synthesis, but less than that with low temperatures and no water stress. For the low temperature trials there were no significant differences in diterpene content between the plants maintained with and without water stress, demonstrating that temperature is the determinant factor in the synthesis of these compounds.

These results are coherent with those obtained under natural conditions, confirming the increase of diterpene in winter, the season when the lowest temperatures are reached. This response of *C. ladanifer* to these climatic factors is the same as that of *Pistacea lentiscus* [24] but different from those of *Pinus halepensis* and *Quercus ilex* [25]. Both Mediterranean species presented positive relationship between total terpenes concentrations and water stress and temperature. These results suggest that secondary metabolism acts differently against environmental changes and depending on the species. Furthermore, the modulation of the amount of the different types of terpenes or other molecules present in plants would be linked to the possible role that they could play in the ecosystem.

Previous studies have demonstrated that phenol biosynthesis in *C. ladanifer* is induced by water stress and high temperatures [11]. In this synthesis, when the plant is subjected to high temperatures, the formation of flavonoids kaempferol-3,7-di(*O*)methyl is favoured [26]. Under natural conditions, the climatic parameters are those of spring and summer, which is when the plant secretes the greatest amounts of flavonoids [11]. While the present results reveal a very different pattern for the synthesis of the diterpenes studied, they reaffirm the idea of the importance of climatic factors in the synthesis of compounds deriving from secondary metabolism. The greatest concentration of phenols occurs in summer and that of diterpenes in winter. Water stress increases the synthesis of phenols but does not determine the greater or lesser presence of terpenes. Temperature is an important modulating factor in the production of terpenes and phenols as it allows the plant to adjust its resistance to different environmental stresses. Such changes in the relative amounts of terpenes and phenols present in leaves under various temperature conditions may also reflect their different functions in the ecology, physiology, and/or biochemistry of the plant in its interactions with microbes, animals, and other plants [27]. In previous studies, it has been shown that two possible functions of the flavonoids in *C. ladanifer* are as a filter of ultraviolet light [8,28] and as a potential defense against herbivory [10]. It has also been demonstrated that the three diterpenes in the *C. ladanifer* exudate, especially diterpenes D1 and D3, inhibit the germination and seedling growth of different herbs [12,16,17], so the results of the present study indicate that diterpenes could significantly contribute to the allelopathy attributed to *C. ladanifer* and in particular for autotoxicity. It has been shown that *C. ladanifer* exhibits autotoxic behaviour during the winter, inhibiting the germination and growth of seedlings that develop during that season [29]. There is further support for the involvement of diterpenes in this process since the rainy season in these zones is autumn-winter [30] and the route of incorporation of these compounds into the soil is via leaching [31], thus providing the highest diterpene concentrations to the soil during the months of autumn [12]. The possibility that these compounds have differentiated functions may be supported by the relationship between their presence and activity in the plant. Thus, the synthesis of flavonoids is enhanced in summer, the season when ultraviolet radiation is the most intense and the plant is very sensitive to herbivore damage. Also, the greatest diterpene concentrations in the leaves correspond to winter, and the diterpenes pass into the soil during that period might interfere in the germination behaviour of the seeds of *C. ladanifer*. Having said that, it is necessary to further deepen in this role and other possible ecological functions of the diterpenes.

## 3. Experimental Section

### 3.1. Selection of Sampling Points and Samples Collection

For this study, four populations of *C. ladanifer* were selected from different points in the province of Badajoz (Extremadura, Spain). The location and climatic characteristics of rainfall and temperature are listed in Table 3. Each population was selected with similar structural characteristics (density, area, age, *etc*.). Four samples of leaves (one per season) were collected throughout the year, over two years. The selected leaves were those born during that same year. The samples were collected from different randomly chosen individuals. All the samples were marked, numbered, and transported in bags to the laboratory for analysis. The rock-rose plants used in the laboratory trials were purchased from a commercial greenhouse. The units were selected at random, all of them with the same structural characteristics (20–30 cm in height) and age (2 years old). They were kept for 2 months under natural conditions before the treatment began.

**Table 3:** Values for the four populations selected for sample collection of: T_max_: mean of the seasonal maximum temperatures (°C); T_min_: mean of the minimum seasonal temperatures (°C); P: total seasonal rainfall (mm). Location UTM coordinates.

		Quintana	Hornachos	Jerez de los	Cabeza
		de la Serena		Caballeros	la Vaca
**UTM coord.**		30 S 262444	29 S 754337	29 S 685866	29 S 728663
		E 4289018	E 4273087	E 4238589	E 4219514
**Spring**	*T_max_* (°C)	25.5	24.2	24.3	25.1
	*T_min_* (°C)	11.3	12.2	11.1	10.9
	*P* (mm)	113	139.8	126	155
**Summer**	*T_max_* (°C)	34.2	32.7	33.3	34.7
	*T_min_* (°C)	18.1	16.3	17.4	18.3
	*P* (mm)	38	44	40	5
**Autumn**	*T_max_* (°C)	16.8	17.8	15.6	15.2
	*T_min_* (°C)	8.4	7.9	8.7	8.8
	*P* (mm)	156	219	300	309
**Winter**	*T_max_* (°C)	14	13.9	14.4	13.9
	*T_min_* (°C)	5.5	6.5	5.2	4.9
	*P* (mm)	130	130	160	187

### 3.2. Laboratory Trial Conditions

Four trials were designed under controlled conditions (in a culture room) of high and low temperatures with and without water stress. All the trials maintained the same photoperiod (14 hours of light). Each trial was conducted on 9 individuals. The conditions of the different trials were the following:

Trial A: High temperatures (30 °C light; 15 °C dark), without water stress (plants watered every day);
Trial B: High temperatures, with water stress (plants moderately watered every 10 days);
Trial C: Low temperatures (13 °C light; 4 °C dark), without water stress;
Trial D: Low temperatures, with water stress.

The temperatures were selected as the means of the summer maxima (30 °C), summer minima (15 °C), winter maxima (13 °C), and winter minima (4 °C) where the distribution of *C. ladanifer* is greatest [32]. Daylight was simulated by visible light lamp Sylvania Gro-lux F30W/Gro-T8. Each experiment was continued for two months. Leaf samples were collected, the exudate extracted and the percentage of wetness quantified, at the beginning and end of each experiment.

### 3.3. Exudate Extraction

Approximately 0.5 g of leaves (2–3 leaves) was dipped several times into 2 mL of chloroform (5 replicates for each determination) [3]. The chloroform was evaporated and the exudates redissolved in 2 mL of methanol, then stored at −20 °C for 12 hours to precipitate out the waxes that were then removed by centrifuging. The supernatant was stored at 4 °C until assay [33].

### 3.4. Sample Analysis; Linear Calibration

**Figure 3:**
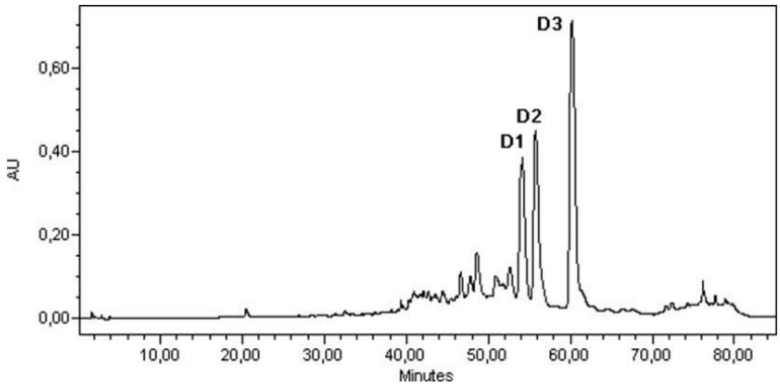
HPLC chromatogram of diterpenes extracts captured at 260 nm. D1: 6-acetoxy-7-oxo-8-labden-15-oic acid; D2: 7-oxo-8-labden-15-oic acid; D3: oxocativic acid.

Quantification of diterpenes in leaf was made by HPLC (Waters, 515 HPLC Pump, 717 plus Autosampler Injector, 996 Photodiode Array Detector). Aliquots of 25 µL were injected into a Spherisorb 5 µ C-18 4.6 × 250 mm reverse phase analytical column. The mobile phase used was water/acetonitrile, with the following gradient: 0–5 min: 100% water (1 mL/min); 5–30 min: 70/30 water-acetonitrile (1 mL/min); 30–65 min: 45/55 water-acetonitrile (1 mL/min); 65–75 min: 100% acetonitrile (1 mL/min); 75–85 min: 100% water (1 mL/min). Once the chromatogram had been obtained (Figure 3), the amount of each diterpene present in the samples was quantified using the corresponding linear calibration equation.

To obtain these equations, the different *C. ladanifer* diterpenes were previously separated and purified by using a semipreparative Nucleosil 5 µ C-18 (250 × 10 mm) column and a water-methanol-tetrahydrofurane (40:30:30) solution at a flow rate of 1.75 mL/min. They were detected with a diode array at 250 nm wavelength. As the peak of each diterpene was detected, it was collected in a separate tube. To eliminate any possible contamination from other compounds eluting close to the diterpene being purified, the fraction was again separated by HPLC with a methanol-water (80:20) solvent at a 2.0 mL/min flow rate. The compounds obtained were identified by comparing their spectral characteristics by NMR with those described in the literature [13,14,15].

Calibration equations:

D1 linear calibration equation: y = 1.1186x; r^2^ = 0.988;
D2 linear calibration equation: y = 0.9307x; r^2^ =0 .998;
D3 linear calibration equation: y = 1.3238x; r^2^ = 0.992.

### 3.5. Statistical Analysis

All variables were tested for normality (Shapiro-Willk test). Parametric tests were used to demonstrate the normality of variables. ANOVA test was used to test differences between seasons and *post hoc* Tukey test was used to compare all pairs of means. Two-way ANOVA test was used to test the influence of temperature and water stress on the amount of diterpene in the leaves. Also, t-Student test for paired samples was used to test the percentage variations in the amount of diterpenes from the beginning to the end of trial. Differences were taken as significant for *p* < 0.05.

## 4. Conclusions

To conclude, we have shown in this study that the amount of flavonoids present in the leaves is markedly seasonal, and it is dependent on climate characteristics, principally the temperature, but in different way than flavonoids. Low temperatures increase the amount of diterpene in the leaves, while higher temperatures increase the amount of flavonoids as demonstrated in previous studies. These differences between the components of exudate suggest the involvement of diterpenes in different ecological functions.
